# Analytical performance evaluation of the Elecsys® Cyclosporine and Elecsys® Tacrolimus assays on the cobas e411 analyzer

**DOI:** 10.1016/j.plabm.2017.03.001

**Published:** 2017-03-10

**Authors:** Maki Sasano, Shigeki Kimura, Ikuhiro Maeda, Yoh Hidaka

**Affiliations:** a Department of Medical Technology, Osaka University Hospital, Japan; b Laboratory for Clinical Investigation, Osaka University Hospital, Japan

## Abstract

**Background:**

Cyclosporine (CsA) and tacrolimus (TAC) are immunosuppressant drugs that are often used to treat autoimmune diseases and as transplantation therapy; therefore, their concentrations need to be monitored carefully. We herein evaluated the analytical performance of the Elecsys® Cyclosporine and Elecsys® Tacrolimus assay kits, which have been newly developed to measure CsA and TAC concentrations in the whole blood.

**Methods:**

We used residual whole blood samples from autoimmune disease and transplantation patients who were being treated with CsA or TAC. CsA concentrations were measured using an affinity chrome-mediated immunoassay (ACMIA) and an electrochemiluminescence immunoassay (ECLIA). TAC concentrations were measured using a chemiluminescence immunoassay (CLIA) and ECLIA. We investigated assay precision, linearity, lower limit of quantitation (LOQ), stability of calibration, influence of interference substances and the hematocrit, correlation of ACMIA with ECLIA, and correlation of CLIA with ECLIA.

**Results:**

Within-assay coefficients of variation were 1.8−3.6% (CsA: 94−1238 ng/mL) and 2.9−3.9% (TAC: 2.1−17.8 ng/mL), whereas day-to-day coefficients of variation ranged between 3.0−4.1% (CsA) and 2.8−3.9% (TAC). The limits of quantitation were defined as the concentration at which the CV was approximately 10%. Each lower LOQ obtained was 16 ng/mL (CsA), and 0.95 ng/mL (TAC). CsA and TAC calibrations were stable for at least 21 days. Neither the presence of conjugated bilirubin, unconjugated bilirubin, chyle, and rheumatoid factor nor the hematocrit affected these assays. A method comparison using a standardized major axis regression analysis of ACMIA and ECLIA was r=0.995, y=0.924x −1.175, n=200 (CsA), while that of CLIA and ECLIA was r=0.994, y=1.080x −0.197, n=200 (TAC).

**Conclusions:**

The analytical performances of the Elecsys® Cyclosporine and Elecsys®Tacrolimus assays were acceptable. Furthermore, CyA and TAC concentrations may be simultaneously measured using a single pretreatment which is of benefit if patients have to undertake conversion between these two drugs. Additionally, it benefits the workflow in the clinical laboratory. Thus, the Elecsys® Cyclosporine and Elecsys® Tacrolimus assays may be suitable for routine therapeutic drug monitoring.

## 1 Introduction

Cyclosporine (CsA) is a calcineurin inhibitor that was first isolated in the 1970s from the fungus *Tolypocladium inflatum*. CsA has a cyclic peptide that consists of 11 amino acids [1]. CsA formulates a complex with cyclophilin in T cells and suppresses interleukin (IL)−2 production by inhibiting calcineurin activity [2]. Tacrolimus (TAC) is a macrolide drug that was isolated from the fermentation broth of a strain of *Streptomyces tsukubaensis*. TAC inhibits the production of T cell-delivered mediators such as IL-2, IL-3, gamma-interferon, tumor necrosis factor (TNF), IL-1β, and IL-6 [3]. Both CsA and TAC are metabolized by CYP3A [4]. P-450-catalyzed reactions are involved in the metabolism of CsA in the human liver. Metabolism is initiated by Cytochrome P-450 of the P-450Ⅲ gene family [5]. Approximately 20 CsA metabolites have been identified and elevated concentrations of these metabolites have been associated with CsA nephrotoxicity [6]. TAC is metabolized by the cytochrome P-450(CYP)3A subfamily and elevated concentrations of TAC have been associated with toxic events. The relationship between the clinical pharmacokinetics of TAC and CYP3A5 genetic polymorphisms has recently been attracting increasing attention [7]. Therefore, in order to reduce the risk of organ rejection and toxicity, CsA and TAC concentrations in whole blood need to be monitored for the effective clinical management of patients receiving CsA or TAC. Whole blood is recommended for measurements of CsA and TAC concentrations because CsA and TAC are incorporated into red blood cells and measurements using whole blood provide better correlations with clinical events than those using plasma [8], [9]. The main analytical methods for CsA and TAC in whole blood have been liquid chromatography combined with mass spectrometric detection (LC-MS/MS), affinity chrome-mediated immunoassay (ACMIA), and chemiluminescence immunoassays (CLIA) [10], [11]. In previous report, each measuring method has difference from LC/MS/MS. Electrochemiluminescence immunoassay (ECLIA) has been shown to have acceptable correlations with LC/MS/MS [12], [13].

We herein report sample pretreatment conditions and analytical performance of ECLIA for detecting CsA and TAC in whole blood using the Elecsys® Cyclosporine and Elecsys® Tacrolimus assay kits with a cobas e411 analyzer.

## 2 Materials and methods

### 2.1 Apparatus

The Elecsys®Cyclosporine and Elecsys® Tacrolimus assay kits were used for measurements of CsA and TAC concentration with a cobas e411 analyzer (Roche Diagnostics GmbH, Mannheim, Germany). ACMIA for measurement of CsA concentration was performed with a Dimension Xpand analyzer (Siemens Healthcare Diagnostics, Inc., IL) and CLIA for measurement of TAC concentration with an ARCHITECT i2000SR (Abbott Laboratories, IL). The hematocrit was assayed using the fully automated hematological analyzer Sysmex XN-1000 (Sysmex Co., Ltd., Kobe, Japan).

### 2.2 Reagent

The Elecsys® Cyclosporine assay kit and the Elecsys®Tacrolimus assay kit were purchased from Roche Diagnostics. The first reagent of these kits contain a cyclosporine-specific or tacrolimus-specific biotinylated antibody, the second reagents contain a ruthenium(Ru)-labeled cyclosporine or tacrolimus derivative, and the microparticle reagents contain streptavidin microparticles.

### 2.3 Sample pretreatment

The pretreatment procedure for whole blood consisted of the precipitation of proteins and extraction of CsA or TAC. The calibrator, controls, and patient samples (300 μL) were combined with 300 μL of the Elecsys ImmunoSuppressive Drug (ISD) Sample Pretreatment Reagent (Roche Diagnostics), a methanol-based solution containing zinc sulfate, in a microcentrifuge tube. The tubes were capped and mixed for 180 s. The MicroMixer E-36 (TAITEC CO., Ltd., Saitama, Japan) was used for sample mixing. Each tube was then centrifuged at 15,700*g* for 4 min in a microcentrifuge. The supernatant was decanted into a sample cup and analyzed with the cobas e411 analyzer.

### 2.4 Assay procedure

In the first step, the pretreated sample (20 μL) was incubated with the CsA- or TAC-specific biotinylated antibody and Ru-labeled CsA or TAC derivative for 9 min. In the second step, streptavidin-coated magnetic microparticles were added and incubated for 9 min. During the second incubation, the entire complex bound to the solid phase through the interaction between biotin and streptavidin. The reaction mixture was aspirated into the measuring cell and the microparticles were attracted to the electrode by magnetic force. Unbound substances were removed by a washing step. Oxidation by charging to the electrode and reduction by tripropylamine induced the chemiluminescent emission of bound substances, which was measured by a photomultiplier. The formation of the respective immune complexes depended on the CsA or TAC concentration in the sample. Sample concentrations were calculated by the emission intensity of the calibrator, which was operated in the same manner [14], [15].

### 2.5 Samples

We used 200 residual EDTA whole blood samples from patients who had received an organ transplant and were under CsA or TAC therapy. The use of patient samples in this study was approved by the Ethics Committee of Osaka University Hospital. We used PreciControl as quality control from Roche Diagnostics GmbH, Mannheim, Germany, Interference check A plus which is component of conjugated bilirubin, unconjugated bilirubin, chyle substances and RF plus to confirm the influence of interference (Sysmex Co., Ltd., Kobe, Japan). Sandimmun^®^ ( Novartis Pharma) or Prograf^®^ ( Astellas Pharma) were used for sample adjustment of the interference study.

### 2.6 Statistical analysis

Data statistical evaluations were performed with Microsoft Office® Excel Professional Edition or SPSS version 19.0 for Windows using a regression analysis or one-way ANOVA. Significance was defined as a P value <0.05. In order to define the relationship and agreement between the Elecsys® reagent based on the ECLIA method and conventional method, a standardized major axis regression analysis and Bland-Altman plots were performed, respectively.

## 3 Results

### 3.1 Sample pretreatment conditions

**Fig. 1:**
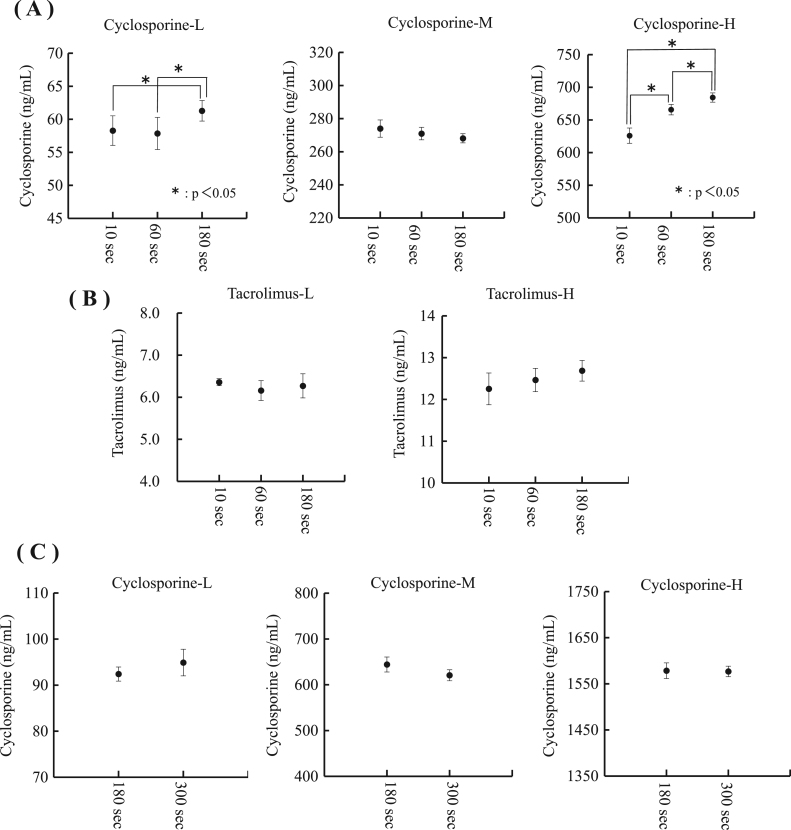
Comparison of sample pretreatment mixing times of 10, 60, and 180 s. Each mixing time and concentration was measured 6 times, and the results were compared by a one-way ANOVA (* p<0.05). (A) Three different CsA concentration (Cyclosporine-L, M, H) samples were compared with mixing times of 10, 60, and 180 s (B) Two different TAC concentration (Tacrolimus-L, H) samples were compared with mixing times of 10, 60, and 180 s (C) Three different CsA concentration (Cyclosporine-L, M, H) samples were compared with mixing times of 180 and 300 s.

It is necessary for accurate measurements of CsA and TAC concentrations to sufficiently mix　samples and pretreatment reagent. The mixing time was evaluated at 10, 60, and 180 s using pooled whole blood samples (CsA: approximately 60 ng/mL, 270 ng/mL, and 680 ng/mL, TAC: approximately 6.0 ng/mL and 12.0 ng/mL). The precipitates of imperfect protein denaturation were confirmed in 10-second mixed CsA and TAC samples that had been pretreated and centrifuged. The measurement of high concentration CsA samples that were mixed for 10 and 60 s revealed significantly lower results than those obtained from samples mixed for 180 s (* p<0.05) (Fig. 1(A)). No significant differences were observed in the mixing times of TAC (Fig. 1(B)). Furthermore, regarding CsA, the mixing time was evaluated at 180 and 300 s using whole blood samples (CsA: approximately 90 ng/mL, 630 ng/mL, and 1570 ng/mL). It was confirmed that no significant differences were noted between mixing times of 180 s and 300 s and over 1000 ng/mL samples (Fig. 1(C)). Based on these results, we decided to mix samples and the ISD pretreatment reagent for 180 s.

### 3.2 Assay precision

The within-assay and day-to-day assay precision for 3 different whole blood controls were tested. Day-to-day assay precision was investigated by performing the assay on 10 days over a two-week period. Duplicate assays were performed each time, and the mean values are shown in Table 1 (CsA) and Table 2 (TAC). The within-assay CV ranged from 1.8% to 3.6% at CsA concentrations between 94 and 1238 ng/mL and 2.1–3.9% at TAC concentrations from 2.1 to 17.8 ng/mL, whereas the day-to-day CV ranged from 3.0% to 4.1% at CsA concentrations between 92 and 1240 ng/mL and 2.8–3.9% at TAC concentrations from 2.0 to 17.5 ng/mL.

**Table 1:** Precision of cyclosporine assay.

**Cyclosporine**	**Mean (ng/mL)**	**SD (ng/mL)**	**CV (%)**
Within-assay ( n=20)			
Control L	94	2.35	2.5
Control M	336	5.90	1.8
Control H	1238	44.66	3.6

Day-to-day assay ( n=10)			
Control L	92	3.76	4.1
Control M	319	12.81	4.0
Control H	1240	37.43	3.0

**Table 2:** Precision of tacrolimus assay.

**Tacrolimus**	**Mean ( ng/mL)**	**SD ( ng/mL)**	**CV (%)**
Within-assay ( n=20)			
Control L	2.1	0.08	3.9
Control M	10.2	0.38	3.7
Control H	17.8	0.37	2.1

Day-to-day assay ( n=10)			
Control L	2.0	0.08	3.9
Control M	10.0	0.29	2.9
Control H	17.5	0.50	2.8

### 3.3 Linearity

CsA and TAC linearities were examined using whole blood samples supplemented with Sandimmun® (Novartis, Basel, Switzerland) or Prograf®Astellas Pharma, Tokyo, Japan). We measured 10-step dilutions of 2000 ng/mL (CsA) and 40 ng/mL (TAC) whole blood pooled samples with the dilution drug-free whole blood. Each assay was performed in duplicate. The dilution curves were linear up to a CsA concentration of at least 2000 ng/mL, and a TAC concentration of at least 40 ng/mL (data not shown).

### 3.4 Calibration stability

**Fig. 2:**
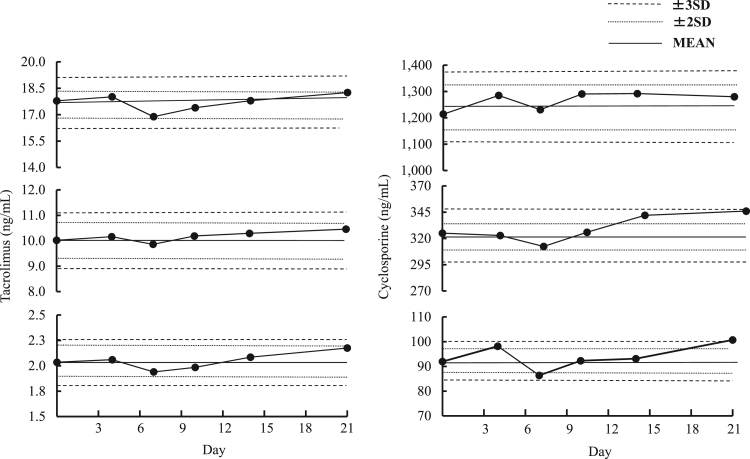
Calibration stability curves. Three different whole blood controls were measured over 21 days. Each assay was performed in duplicate, and mean values were within variation ranges of the day-to-day assay precision.

We examined the calibration stability by measuring 3 different whole blood controls over 21 days. Each assay was performed in duplicate, and mean values were within the variation range of the day-to-day assay precision. CsA and TAC calibrations were both found to remain stable for 21 days (Fig. 2).

### 3.5 Lower limit of quantitation

**Fig. 3:**
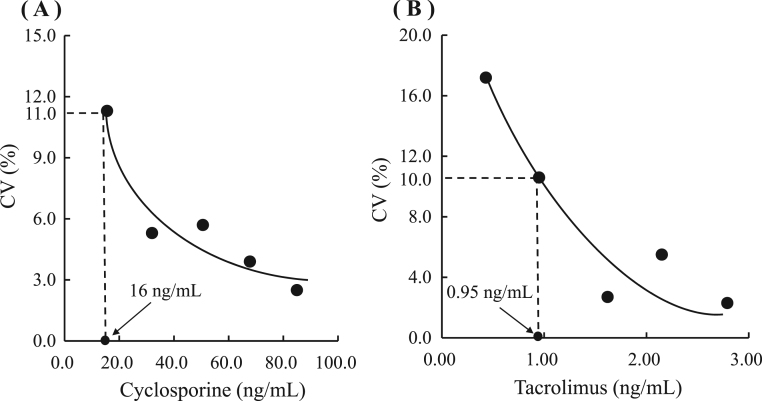
Limit of Quantitation (A) We measured serial dilutions of 85 ng/mL (CsA) whole blood pooled samples with the dilution drug-free whole blood on day 5. Each assay was performed in triplicate, and the LOQ was defined as the concentration at which the mean CV was approximately 10%. (B) We measured serial dilutions of 2.7 ng/mL (TAC) whole blood pooled samples with the dilution drug-free whole blood on day 5. Each assay was performed in triplicate, and the LOQ was defined as the concentration at which the mean CV was approximately 10%.

We measured serial dilutions of 85 ng/mL (CsA) and 2.7 ng/mL (TAC) whole blood pooled samples with the dilution drug-free whole blood for five days running. Each assay was performed in triplicate and the LOQ was defined as the concentration at which the CV was approximately 10%. Each lower LOQ obtained was 16 ng/mL (CsA), and 0.95 ng/mL (TAC) (Fig. 3).

### 3.6 Influence of interference substances and the hematocrit

**Fig. 4:**
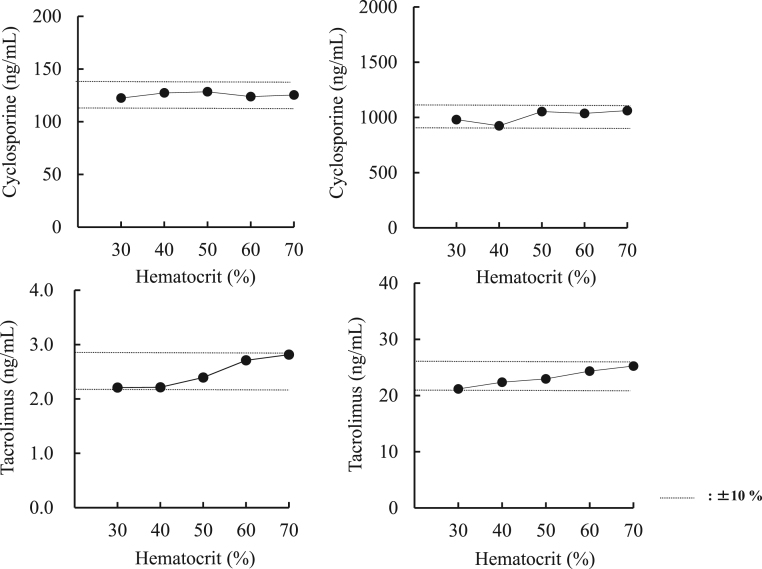
Effects of the hematocrit on CsA and TAC assays using the ECLIA method. The hematocrit in blood samples was adjusted to 30%, 40%, 50%, 60%, and 70%. We added Sandimmun® or Prograf® was added to each hematocrit sample and examined the effects of the hematocrit at two different concentrations of CsA and TAC.

There was no significant interference within the recovery rate of±10% which was caused by the addition of 211 mg/L conjugated bilirubin, 211 mg/L unconjugated bilirubin, 2800 IU/L　rheumatoid factor or 1490 formazine turbidity units of chyle material to control whole blood pooled samples. The effects of the hematocrit on this assay for CsA and TAC were also evaluated by using whole blood samples from healthy volunteers. The hematocrit was assayed using the fully automated hematological analyzer Sysmex XN-1000 (Sysmex Co., Ltd., Kobe, Japan). The hematocrit of the blood samples was adjusted to 30%, 40%, 50%, 60%, and 70%. After each blood sample was centrifuged, plasma were removed. Then, by adding the same amount of Sandimmun® or Prograf® as the removed plasma, we examined the series with different hematocrit values with constant concentrations of CsA and TAC. Variations in the hematocrit up to 70% had minor insignificant influence (Fig. 4).

### 3.7 Method comparisons

**Fig. 5:**
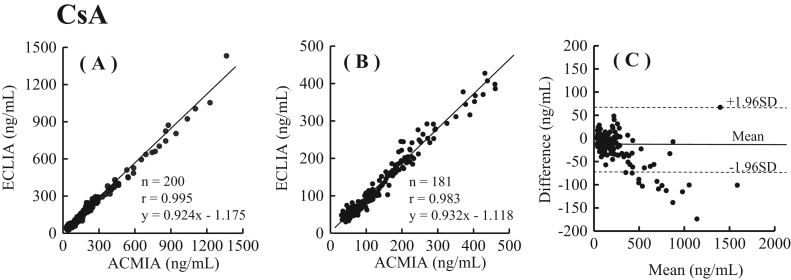
Comparison of whole blood CSA concentrations measured by the Elecsys Cyclosporine assay and ACMIA method. (A) A standardized major axis regression analysis (CSA: 0 – 1500 ng/mL): Elecsys Cyclosporine assay (y) versus the ACMIA method (x). (B) A standardized major axis regression analysis (CSA: 0 – 500 ng/mL): Elecsys Cyclosporine assay (y) versus the ACMIA method (x). (C) A Bland-Altman plot showing the difference between CsA values obtained with the Elecsys Cyclosporine assay and ACMIA method. The mean difference (solid line) and±1.96 SD of the mean difference (dotted line) are shown parallel to the x axis.

**Fig. 6:**
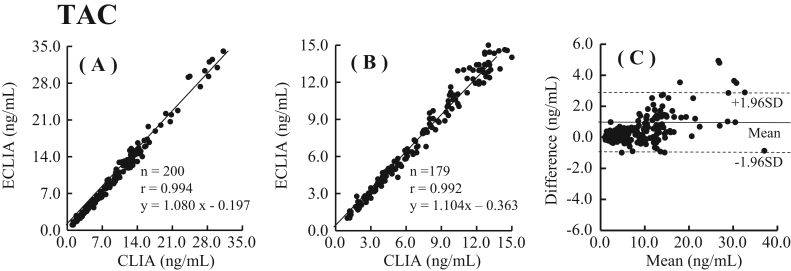
Comparison of whole blood TAC concentrations measured by the Elecsys Tacrolimus assay and CLIA method. (A) A standardized major axis regression analysis (TAC: 0 – 35 ng/mL): Elecsys Tacrolimus assay (y) versus the CLIA method (x). (B) A standardized major axis regression analysis (TAC: 0 – 15 ng/mL): Elecsys Tacrolimus assay (y) versus the CLIA method (x). (C) A Bland-Altman plot showing the difference between TAC values obtained with the Elecsys Tacrolimus assay and CLIA method. The mean difference (solid line) and±1.96 SD of the mean difference (dotted line) are shown parallel to the x axis.

We examined the relationship between CsA values obtained by the Elecsys® Cyclosporine assay and data obtained with ACMIA CsA by a standardized major axis regression analysis. We also examined the relationship between TAC values obtained by the Elecsys® Tacrolimus assay and data obtained with the CLIA TAC assay in the same manner. Furthermore, we compared differences between the Elecsys® Cyclosporine assay and data obtained with ACMIA CsA, and between the Elecsys® Tacrolimus assay and data obtained with the CLIA TAC assay using the Bland-Altman technique. Each regression line is shown in Figs. 5A and 6A. The correlation of CsA assay between ACMIA and ECLIA was r=0.995, y=0.924x −1.175, n=200, while that of TAC assay between CLIA and ECLIA was r=0.994, y=1.080x −0.197, n=200. When the extent of the agreement between the methods was assessed using the Bland-Altman technique (Figs. 5B, 6B), the mean difference was −17.51 (95% limits of agreement, −78.37 to 60.85, all data) for the Elecsys® Cyclosporine assay versus ACMIA, whereas it was 0.51 for the Elecsys® Tacrolimus assay versus CLIA (95% limits of agreement, −1.30−1.30 to 1.81). However, the Elecsys® Cyclosporine assay showed lower concentrations than ACMIA when samples containing more than 500 ng/mL CsA were examined.

## 4 Discussion

In the present study, we evaluated the analytical performance of the Elecsys® Cyclosporine and Elecsys® Tacrolimus assays. Since CsA and TAC are largely distributed in red blood cells and bound to proteins, a one-step manual pretreatment is performed to release the analytes from the proteins [16], [17]. This step is very important for accurate measurements of CsA and TAC concentrations. Regarding the mixing time of samples and the pretreatment reagent, this was only described as “vortex” in the package leaflet. We compared mixing times of 10, 60, 180, and 300 s. We demonstrated that a pretreatment mixing time of more than 180 s is necessary in order to obtain stable results. The Elecsys® Tacrolimus concentrations were elevated at higher hematocrit values. A previous study indicated that negative correlations between the hematocrit value and tacrolimus concentration were caused by Micro Particle Enzyme Immunoassay optics [16]. However, our results were completely the opposite. TAC is distributed in red blood cells more than CsA; therefore, high hematocrit values have no effect on measurements of CsA concentrations, but influence those of TAC concentrations. Concerning the influence of the hematocrit, if TAC is completely extracted from samples, its concentration at a high hematocrit may be greater than that at a low hematocrit. Since we did not examine the influence of the hematocrit using completely hemolytic samples in the present study, the cause for this remains unclear; but it was not considered to be a problem, because this tendency is within a reproducible error range.

The Elecsys®Tacrolimus assay showed good precision with a reasonable LOQ, good linearity, no interference except hematocrit, and its correlation with CLIA methods was also good. The Elecsys® Cyclosporine assay showed good precision with a reasonable LOQ, good linearity, and no interference. However, the Elecsys® Cyclosporine assay showed lower concentrations than ACMIA methods when samples containing more than 500 ng/mL CsA were examined. This result may be attributed to the measuring mode. The linearity of ACMIA method is 500 ng/mL, we measured over 500 ng/mL samples with the high concentration measurement mode “CSAE”, which is the reagent for measuring high concentration of CsA (500−2000 ng/mL). If measurement is performed using reagents immediately after dissolution of the reagent, there is a possibility that the measurement result may fluctuate largely. Thus, our results may be explained by the CSAE mode, although the exact cause remain unclear. In the ACMIA method and the ECLIA methods, a slight difference occurs in the measurement result due to the difference in reactivity of the antibody contained in the reagent. As a result, there was a possibility that significant difference was observed in the high concentration range. We consider that further verification of the difference in reactivity of the antibodies in each method is necessary.

In conversion between TAC and CsA, the simultaneous measurement of TAC and CsA concentrations is necessary in order to estimate suitable doses and target trough levels [17]. Elecsys® Cyclosporine and Tacrolimus can simultaneously measure the concentration of tacrolimus and cyclosporine in the blood at a single pretreatment and the measurement time is shortened by the current method ACMIA method and CLIA method. So, the Elecsys® Cyclosporine and Elecsys® Tacrolimus assays may be suitable for routine therapeutic drug monitoring.

## 5 Conclusion

Although care should be taken when measuring patient samples with high CsA concentrations, CsA and TAC concentrations may be simultaneously measured using a single pretreatment. Furthermore, the measuring time of the Elecsys® Tacrolimus assay is shorter than that of CLIA methods. The results of the present study indicate that the Elecsys® Cyclosporine and Tacrolimus assays correlate well with established methods, that the LOQ and linearity are acceptable for use in the patient population to be tested and that the precision is clinically acceptable.
